# Importance of molecular cell biology investigations in human medicine in the story of the Hutchinson-Gilford progeria syndrome

**DOI:** 10.2478/v10102-010-0018-y

**Published:** 2010-09

**Authors:** Ivan Raška

**Affiliations:** Charles University in Prague, First Faculty of Medicine, Institute of Cellular Biology and Pathology, and Institute of Physiology, Academy of Sciences of the Czech Republic, v.v.i., Department of Cell Biology, Albertov 4, 128 01, Prague, Czech Republic

**Keywords:** Laminopathies, Hutchinson-Gilford progeria syndrome, lamin A, progerin

## Abstract

Ranged among laminopathies, Hutchinson–Gilford progeria syndrome is a syndrome that involves premature aging, leading usually to death at the age between 10 to 14 years predominatly due to a myocardial infarction or a stroke. In the lecture I shall overview the importance of molecular cell biology investigations that led to the discovery of the basic mechanism standing behind this rare syndrome. The genetic basis in most cases is a mutation at the nucleotide position 1824 of the lamin A gene. At this position, cytosine is substituted for thymine so that a cryptic splice site within the precursor mRNA for lamin A is generated. This results in a production of abnormal lamin A, termed progerin, its presence in cells having a deleterious dominant effect. Depending on the cell type and tissue, progerin induces a pleiotropy of defects that vary in different tissues. The present endeavour how to challenge this terrible disease will be also mentioned.

## Introduction

I work in the field of molecular cell biology and the projects of our laboratory are distant from pharmacology and toxicology. Therefore, instead of showing you results from some work originating from our laboratory (see kindly “http://lge.lf1.cuni.cz”), I shall tell you a story of the Hutchison-Gilford progeria syndrome (HGPS) that explicitly documents the importance of molecular cell biology investigations in human medicine. In order to fulfill this aim simply, I explain here the results of only a few of the relevant experiments that are directly linked to the HGPS story.

The HPGS is one type of laminopathies, alternatively called the nuclear envelope diseases. Most cases of HGPS result from a nucleotide position 1824C → T mutation (substitution mutation from cytosine to thymine) in the gene coding lamin A (*LMNA*) that creates an ectopic mRNA splicing site leading to an expression of truncated prelamin A. If compared to “normal” prelamin A protein, truncated prelamin A lacks 50 amino acids within its tail domain. Both normal and truncated prelamin A proteins are subjects to extensive post-translational modifications (PTMs). The PTMs play an important role in the HGPS story.

Generally speaking, it should be noted here that the causes of many human diseases are due to an incorrect splicing of various precursor mRNAs.

## Organization of the nuclear envelope

The nucleus of human cells (and of eukaryotic cells in general) is delimited by a nuclear envelope (NE) formed by two membranes, the inner nuclear membrane (INM) and the outer nuclear membrane (ONM). Importantly, the NE keeps separate most of the genome (= nuclear DNA) as well as other nuclear components (such as some enzymes) separated from cytosolic components. The nucleus is an important example of cellular compartmentalization that allows the cell to properly function. Biochemical reactions are facilitated by the high concentration of both substrates and enzymes within cell compartments.

The nuclear envelope is “punctured” by large nuclear pore complexes (NPCs) at sites where the ONM and INM join. The NPCs serve for a transport of (macro)molecules between the nucleus and the cytosol (cytoplasm). The nuclear envelope is, via the ONM, directly connected to the membranes of the endoplasmic reticulum. The INM is structurally supported by a nuclear lamina, the core of which consists of a network of intermediate filaments (IF) composed of lamin proteins belonging to the family of intermediate filament proteins. Many other proteins are associated with the nuclear lamina, such as heterochromatin protein 1 (HP1), which provides a link between the lamina and chromatin, or the protein BAF (barrier to autointegration factor) which is a part of the protein complex linking the INM with the lamina.

Numerous integral membrane proteins are embedded in both ONM and INM and exhibit lateral movements within the membrane. Some proteins anchored within the ONM associate with cytoplasmic cytoskeleton (*e.g.* actin and intermediate filaments). Moreover, some proteins anchored within the INM can interact with proteins within the ONM. Many of those proteins in the NE physically interact, either directly or via a protein complex, with the nuclear lamina that underlies the INM, and are called lamin-associated polypeptides (LAPs; one such protein is BAF).

## A few words on splicing

The first steps of gene expression occur in the cell nucleus. Typically, the synthesis and processing of precursor mRNA (pre-mRNA) take place there.

A gene is transcribed and this process gives rise to a primary transcript, a pre-mRNA. This precursor molecule is then processed. I mention here three major processing (“maturation”) events:

- capping – chemical modification of the 5′end of the pre-mRNA and generation of the “cap” structure;
- polyadenylation – the terminal 3′end of pre-mRNA is cleaved and (tens of) adenines are added at the 3′end;
- splicing – a process during which non-coding sequences (introns) are cleaved away from pre-mRNA and remaining coding sequences (exons) are joined (ligated) together.

The mature mRNA is then transported into the cytoplasm and its sequence is translated on ribosomes into a polypeptide chain.

Splicing is a complicated process that involves many splicing factors and a number of small nuclear ribonucleoproteins (snRNPs) containing small nuclear RNAs (snRNAs). Standard snRNPs contain snRNA of type U1, U2, U4, U5 or U6 (the letter U was used due to the high content of uridine in this type of RNA).

There are special consensus nucleotide sequence signals within the pre-mRNA that are necessary for the binding of snRNPs and variety of splicing factors. Among them, there is a consensus sequence at the beginning of intron sequence that is necessary for the binding of U1 snRNP. Within this sequence, a consensus triplet GGU is found that plays a prime role in the story of HGPS.

This being said, it is also necessary to review the process of alternative splicing. Depending mainly on the cell type and the tissue, the fate of a given pre-mRNA may vary as different exons can be used in the generation of mature mRNA. This is well documented in the example of the mouse α-tropomyosin gene. For instance, in striated muscle cells, exon 1 and 3 are found in mature mRNA, but not exon 2. In contrast, in smooth muscle cells, exon 1 and 2 are found in mature mRNA, but not exon 3. Accordingly, different α-tropomyosin molecules are synthesized in striated or smooth muscle. An analogous situation occurs in human cells which contain about 25000–30000 genes, but through alternative splicing many more (usually similar) proteins are found in the human body. The alternative splicing of pre-mRNA for lamin A/C is important for the HGPS story.

## Lamins

Human cells contain two lamin genes, the gene for lamin B and the gene for lamin A/C (*LMNA*). Lamins B are expressed in most cells in both embryos and adults, and its expression is essential for nuclear integrity, cell survival, and normal development. In contrast, lamins A/C are differentially expressed, and their appearance in any cell type is normally correlated with differentiation. Lamins A and lamin C are just splice variants. The lamin A/C gene pre-mRNA contains 12 exons, and lamin A and C mRNAs are generated via alternative splicing of the same pre-mRNA. For the HGPS story, only the lamin A is relevant since mature lamin C mRNA does not contain exons 11 and 12.

The contrasting expression patterns of lamins B and A/C, together with the finding that B-type lamins are essential for cell survival, have given rise to the notion that B-type lamins are the fundamental building blocks of the nuclear lamina, while A-type lamins have more specialized functions.

## Laminopathies

Mutations in genes coding for lamins and LAPs give rise to a pleiotrophy of human diseases called laminopathies, also called nuclear envelope diseases. The great puzzle is that various tissues are affected in patients, and the diseases exhibit clinical variations and a genetic heterogeneity. Not only *LMNA*, which has over 200 mapped mutations, but also emerin (EMD), the zinc protease (metallopeptidase) ZMPSTE24 (Face1), lamin B receptor (LBR), SUN2 (integral transmembrane INM protein that associates with Nesprin within the lumen of the NE), Nesprin, Torsin A, MAN1 (one of the three membrane proteins originally identified by human autoantibodies from a patient suffering from a collagen vascular disease) and many other LAPs may bear mutations.

Hundreds of proteins are associated with the NE. Depending on the cell type and the tissue, the proteome of the NE (*i.e.* proteins associated with NE/nuclear lamina) differ. Therefore, the genetic heterogeneity apparently plays a role in various manifestations of laminopathies as multiple interacting proteins cause variants of the disease (depending on a given mutation, a disruption of tissue specific complexes associated with NE/nuclear lamina may lead to various disease manifestations).

*Speaking about the importance of molecular cell biology investigations for human medicine, here already is an important message: human diseases have origin in the changed functioning of cells that may manifest itself differently in various cell types and tissues.*

This being said, let us begin with the HGPS story and focus on a *de novo* acquired mutation in the nucleotide position 1824 of the LMNA gene sequence.

## The HGPS story

After birth, children bearing such a *de novo* acquired mutation have no symptoms of the disease. The disease onset is situated between 12 and 24 months after birth, with a life expectancy of 10 to 15 years. HGPS exhibits many features such as accelerated aging, cardiovascular defects, atherosclerosis, sclerotic skin, joint contractures, bone abnormalities, alopecia and growth impairment. Various tissues are affected differently, but children with HGPS have about normal cognitive and other brain functions so that the kids are aware of their “special” situation. The kids usually pass away due to a myocardial infarction or a stroke. Fortunately, this disease is very rare and only 1 in 4 millions children are affected.

Molecular cell biology helped to decipher the molecular cause standing behind the disease. I document here results of an important experiment. Small fragments of the skin were taken from HGPS and healthy (control) children, and respective skin fibroblasts were cultured. The fibroblasts were stained with DAPI, a fluorescent dye staining DNA, and labeled for LMNA with specific antibodies to lamin A. There is a striking difference in the appearance of nuclei. Most of the normal fibroblasts exhibit a “smooth” appearance of nuclei. In contrast, most of the diseased nuclei exhibit irregular shapes with inward facing protrusions (wrinkles). Clearly, the nuclear lamina was involved. What was behind this?

The LMNA gene from children with HGPS was sequenced and it was established that in one of the two LMNA genes (human cells are diploid, therefore two chromosomes bear the LMNA gene), there is a mutation in the 11th exon at the nucleotide position 1824. At this position, T was substituted for C. With such a mutation, one would expect that nothing happens, as both of the coding triplets GGC and GGT code for the amino acid glycine (at the protein level: Gly608Gly). However, a GGT sequence in DNA becomes a GGU sequence in the transcribed pre-mRNA. This generates a new (cryptic) splicing site, as the GGU triplet is important for the binding of U1 snRNP.

Messenger RNA of lamin A was analyzed by electrophoresis in gels and lamins A and lamin C analyzed by Western blotting in which detection of lamins A/C was performed with an antibody reacting both with lamin A and lamin C. Messenger RNA from the HGPS child exhibited two bands in the gels, one for normal lamin A, and an additional band that was shorter by 150 nucleotides. This was due to the aberrant splicing of pre-mRNA. At the protein level, bands for both lamin A and lamin C were seen, but an additional reactive band was also seen that was shorter by 50 amino acids with respect to lamin A (150 nucleotides in mRNA correspond to 50 amino acids in protein). It corresponded to the aberrant smaller lamin A protein, designated Δ-lamin A. This Δ-lamin A protein was later called progerin.

How does the HGPS mutation cause the diseased cell phenotype? There are two explanations possible. Either the level of normal lamin A protein is insufficient to maintain the normal nuclear lamina function, causing a haplo-insufficiency effect, or the mutant protein disrupts normal lamina function, which would be considered a dominant negative effect.

Cell biology again helped to settle this problem. Diseased and normal fibroblasts were transfected with one of two plasmids. One carried the sequence coding for normal lamin A, the other for Δ-lamin A. These sequences were ligated to the sequence coding for green fluorescent protein (GFP). Bearing in mind that not all cells were successfully transfected and allowed for the expression of recombinant proteins lamin A-GFP or Δ-lamin A-GFP, the results of these experiments were clear cut. If progeric fibroblasts were transfected with lamin A-GFP plasmid, the abnormal nuclear phenotype was not rescued after transfection. This meant that additional “normal” lamin A proteins did not rescue the phenotype. Therefore, haplo-insuficience is not valid. In contrast, if healthy cells were transfected with Δ-lamin A-GFP, the nuclei of transfected cells exhibited an abnormal shape. Thus, the dominant negative effect applies and the presence of progerin is the cause of the disease phenotype.

An experiment using fluorescence recovery after photobleaching (FRAP; a method in optical microscopy for determining the mobility of proteins within a cell-a rationale of this method is explained in the reference by Lippincott-Schartz & Patterson, 2003) brought another important cell biology result. In this experiment all combinations of healthy or HGPS cells and transfections with lamin A-GFP or Δ-lamin A-GFP plasmids were assayed. An important message followed from this experiment - progerin was “cemented” in the nuclear lamina, and the cell was accumulating this aberrant protein. Moreover, the exchange of normal lamin A molecules within the nuclear lamina was lowered. The mechanical properties of the NE were also changed, for example the nuclei of diseased cells were often ruptured during nuclear microinjection experiments. Thus, it seems that the nuclear envelope is much more “stiff” in cases of HGPS.

In summary, HGPS is not due to haplo-insufficiency, as reintroduction of additional “wild type” lamin A is insufficient for rescue. In contrast, the progerin itself generates the abnormal cell phenotype, it accumulates in the lamina, and the dynamics of the nuclear membrane are decreased.

Is it possible to rescue the phenotype of the diseased cells, and eventually help the children with HGPS? There is no way to repair the deleterious mutation throughout the whole human body. Remaining potential possibilities are at the level of the aberrant mRNA and/or progerin.

## Reversal of the HGPS cellular phenotype at the mRNA level

It is indeed possible to eliminate the aberrant LMNA mRNA in HGPS cells cultured *in vitro*. For this purpose, short complementary antisense morpholino oligonucleotides (morpholino oligonucleotides have a chemical structure that is resistant to nucleases) to aberrant pre-mRNA were synthesized. The sequence of oligonucleotides encompassed the nucleotide 1824 mutation site.

The oligonucleotides were introduced into diseased cells and bound at the mutation site of most abberant pre-mRNAs. The splicing machinery could not function because U1 snRNP could not bind to the cryptic splicing site. As a net result, basically just the normal splicing could occur in the cell. The result at the cell level was excellent: while HGPS cells exhibited 70% of aberrant nuclei, such cells after treatment with antisense oligonucleotides exhibited only 10% of aberrant nuclei, *i.e.* about the same number as healthy (control) cells exhibiting 7% of aberrant nuclei.

Via inhibitory oligonucleotides, one could indeed experimentally rescue the cell phenotype in cells cultured *in vitro*. However, within the human body, no convenient way to administer such oligonucleotides to all cells is known.

Is there a way, at the level of progerin, to improve the fate of children suffering from HGPS? Before replying, I have to tell you about the PTMs of prelamin A and progerin.

## Posttranslational modifications of prelamin A and progerin

Messenger RNA for lamin A is translated, giving rise to the precursor protein “prelamin A,” which is subject to extensive PTMs. One of the modifications is addition of a farnesyl group to prelamin A. At the C-end of normal (as well as progeric) prelamin A, the polypeptide chain has a special sequence of 4 amino acids, cysteine-serine-isoleucine-methionine (CSIM) sequence. This sequence, termed generally a „CAAX box“, is the target site of an enzyme – farnesyl transferase (FTase). The enzyme adds to the cysteine of the CSIM sequence a lipophilic farnesyl group. Cysteine farnelysation results in the association of prelamin A as well as progerin with the (lipophilic) INM. It should be noted that farnesylation of proteins is a common feature of numerous proteins containing CAAX box sequences (*e.g.*, Ras protein is farnesylated).

In the cell, still other modifications of prelamin A take place. For example, the sequence SIM is cleaved away and is replaced by carboxymethylation of cysteine. However, the important modification for HGPS is that the terminal sequence in prelamin A is cleaved by the endoprotease Zmpste24. This protease cleaves off the end of the prelamin A sequence, including the farnesylated cysteine, and gives rise to mature lamin A protein. Lamin A is thus no longer targeted to the INM. Importantly, this cleavage cannot take place in the progerin molecule as the endoprotease needs the missing 50 amino acids to perform the cleavage job. As the results, progerin remains anchored to the INM and accumulates there.

In summary, farnesylation of progerin has a deleterious effect on the diseased cell.

And importantly, I documented here how molecular cell biology helped to discover the basic mechanism behind the HGPS.

## Reversal of the HGPS cell phenotype and initial clinical trials with inhibitors of farnesyl transferase

How about blocking the farnesylation of progerin? Inhibitors of the farnesyl transferase are known (*e.g.* the drugs Tipifarnib and Ionafarbid) and can be administered to patients similarly as standard drugs. At the cell level, the aberrant nuclear form seen in progeric cells can be rescued by FTase inhibitors.

Using FTase inhibitors, very promising experimental results have been obtained with mice exhibiting a mutation in the LMNA gene and manifesting HGPS-like syndrome. I shall document how the histologic picture of the descending aorta of 9-12 month old progeric mice is improved. Daily administration of inhibitor prevented the loss of the vascular smooth muscle cells seen in non treated progeric animals.

Highly demanding clinical treatments have now begun at Harvard University (USA) with HGPS children from all over the world. It is to be emphasized that other cellular processes are affected by the FTase inhibitor treatments because, besides progerin, many other proteins need to be farnesylated in order to perform their physiological function. In other words, the administration of FTase inhibitors necessarily exhibits toxic effects. When the toxic effects manifest themselves, the treatment of the children with HGPS is discontinued until manifestations of toxicity disappear, then the administration of the drug is resumed. Personally, I am anxious to hear positive reports concerning this terrible disease.

Experimental and clinical investigation of the Hutchinson–Gilford progeric syndrome is potentially important from another point of view. It has been shown that some accumulation of progerin takes place in normal human cells during aging (the cell is not a robot and accumulating failures occur in the cells). Despite the fact that children with HGPS have about normal brain function while central nervous system degeneration accompanies normal human aging, progress in our knowledge of HGPS may bring some important clues about the mechanisms of normal aging as well.

And I hope that, with the HGPS story, I was able to document the importance of molecular cell biology investigations in human medicine.
